# Associations of Cognitive Function with BMI, Body Fat Mass and Visceral Fat in Young Adulthood

**DOI:** 10.3390/medicina55060221

**Published:** 2019-05-28

**Authors:** Tao Huang, Zuosong Chen, Liqun Shen, Xiang Fan, Kun Wang

**Affiliations:** Department of Physical Education, Shanghai Jiao Tong University, Shanghai 200240, China; taohuang@sjtu.edu.cn (T.H.); zschen1971@126.com (Z.C.); shenliqun911@163.com (L.S.); fansheva@sjtu.edu.cn (X.F.)

**Keywords:** body composition, obesity, cognitive function, executive function, adulthood

## Abstract

*Background and objectives*: Existing studies concerning the associations of cognitive function with adiposity in young adults are sparse. The purpose of the study was to examine the associations of adiposity with cognitive control in young adults. *Materials and Methods:* Participants were 213 young adults (98 women and 115 men). Cognitive control was measured using a modified task-switching paradigm. Anthropometrics were measured by standardized procedures. Body fat mass and visceral fat area were measured by bioelectrical impedance analysis. *Results*: The results showed that increased body mass index (BMI, *p* = 0.02), body fat percentage (*p* = 0.02), and visceral fat area (*p* = 0.01) were significantly correlated with larger global switch costs of accuracy in women. In men, high levels of body fat percentage (*p* = 0.01) and visceral fat area (*p* = 0.03) were significantly correlated with larger local switch costs of reaction time. *Conclusions*: The results indicated that elevated adiposity was associated with worse performance on measures of cognitive control in young adults.

## 1. Introduction

With its increased prevalence over the past three decades, obesity has become a public health problem in many countries [1,2]. In China, it was estimated that the rate of overweight and obesity in adults was 41.2% and 12.9% in 2014 [3], respectively. It is well-documented that higher body mass index (BMI) results in an increased risk of many chronic diseases, such as cardiovascular diseases and strokes [4,5]. Moreover, higher BMI increased the risk of all-cause mortality [6]. Even in young adulthood, higher BMI causes worse cardiovascular health, such as higher blood pressure and left ventricular mass index [7]. In addition, recent evidence has linked obesity with the brain and cognitive functions [8]. Several longitudinal studies have demonstrated that excessive body fatness in adulthood is prospectively associated with increased risks of cognitive dysfunction and dementia in later life [9,10,11]. Although the potential biological pathways underlying the associations are not fully understood, existing evidence from clinical studies and animal studies have suggested some potential risk factors which may be involved, such as impaired insulin and glucose regulation [12,13], increased systemic and central inflammation [8,14], and increased brain atrophy [15].

Notably, in the aforementioned studies examining the associations of obesity with cognitive function, the study populations were primarily older adults or middle-aged adults. Considering the abovementioned risk factors are less prevalent in healthy young adults [16,17], it is of importance to clarify whether or not the impact of excessive body fat on cognitive function is evident in young adulthood. However, only a few of studies concerned the associations of adiposity with cognitive function in this age group. Recently, a negative association of obesity with cognitive function in young adulthood was reported in the domain of cognitive control. Cognitive control, also known as executive function, refers to a set of cognitive skills used for controlling and coordinating other cognitive abilities and behaviors. Cognitive control receives increasing attention of research due to its relationship with many aspects of daily life, such as health behaviors, cognitive, social, and psychological developments [18,19]. Cognitive control consists of three core functions (i.e., inhibition control, working memory, cognitive flexibility) and higher-level functions, such as planning, reasoning, decision-making, etc. [18]. Song et al. [20] showed that, in young college students, obese and low-fitness individuals had worse inhibitory control as measured by a Stroop color and word test. Similarly, Catoira et al. [21] found that young women with obesity had worse inhibitory control compared with their normal weight counterparts. However, those studies only compared inhibitory control between obesity group and normal weight counterparts. Therefore, it is still not clear whether or not there is a linear association between cognitive control and adiposity. Furthermore, the potential associations of adiposity with two other core domains of cognitive control, namely working memory and cognitive flexibility, have yet to be elucidated.

The present study aimed to investigate the associations of three indicators of adiposity (i.e., BMI, body fat percentage, and visceral fat area) with performance on a task-switching paradigm which requires working memory and cognitive flexibility in healthy young adults.

## 2. Materials and Methods

### 2.1. Study Participants

The participants were randomly recruited from a University in Shanghai, China. The students were advised the opportunity of participation in the study by their teachers and by advertisements on the campus. Healthy college students without physical and mental conditions were eligible for participating in the study. Participants were excluded if they denied the measures or they could not take part in the bioelectrical impedance analysis-based body composition measurements, such as wearing metal medical devices (e.g., artificial tooth with metals). Two hundred and twenty-two female and male college students volunteered to participate in the study. The measurements were conducted from May to December 2016. Of the participants, nine did not complete the measurements on body composition and/or cognitive control due to personal reasons (i.e., refusing to participate in the measurements). Therefore, 213 participants who completed the measurements on adiposity and cognitive control were included in the analyses of this study. Informed consents were obtained from the participants. All measures were scheduled on one testing day (in the morning or in the afternoon). The participants first completed the questionnaire. Afterwards, they took part in the body composition measurement and cognitive measures in a random order. The study protocol was approved on January 10, 2017, by the ethical committee at Shanghai Jiao Tong University (approval No:20170100).

### 2.2. Anthropometrics and Body Composition

Body height was assessed with bare feet using a wall mounted stadiometer (Seca 206, Seca, Hamburg, Germany). Participants’ body composition and body weight were assessed using an Inbody 720 Body Composition Analyzer (Inbody Co. Ltd., Seoul, Korea). The bioelectrical impedance analysis-based analyzer provides valid and rapid estimation on total and regional body composition [22,23]. Participants wore light clothing with bare feet on the analyzer. All metal items were removed before the analysis. Body fat percentage (%) and visceral fat area (cm^2^) were used in this study. BMI was defined as body weight (kg) divided by square of height (m). BMI cut-off points for overweight and obesity were defined as: Overweight, BMI = 24.0–27.9 kg/m^2^; obesity, BMI ≥ 28 kg/m^2^ [24].

### 2.3. Cognitive Control

A widely used task-switching paradigm was used to assess cognitive control [25]. The measures of task-switching test reflect participants’ working memory and cognitive flexibility. The stimuli were presented via the Windows-operated E-Prime 2 professional. The cognitive testing was administered individually in comfortable testing environment without distraction. Before conducting cognitive measures, the participants were instructed to sit down and rest for 10 min.

Eight numeric digits (1–9, excluding 5) surrounded by either a solid square or a dashed square were presented as stimuli. The white-colored stimuli were randomly presented with equal probability for 200 ms in a black background, and the interval between each stimulus was 1500 ms. There are two tasks cued by the square. Specifically, when the number appeared with a solid square, the task is to judge if the number is greater or smaller than five (high/low trials). When the number appeared with a dashed square, the task is to judge if the number is even or odd (odd/even trials). There are two pure blocks (one high/low trial block and on odd/even trials block) and four mixed blocks in the paradigm. Each block consisted of 64 trials. In the mixed blocks, every two high/low or odd/even trials were presented in a predictable alternating runs paradigm [26]. Therefore, an equal number of switch and repetition trials were included in the mixed blocks. The participants were instructed to response as quickly and accurately as possible using a keyboard. When the digits are smaller than five or odd, the response key was set as X. When the digits are greater than five or even, the response key was M. The trials are terminated 3000 ms after onset if there are no responses. The participants were instructed to complete 64 practice trials before the pure and mixed blocks to ensure the understanding of the task instruction.

Raw data were screened for outliers, where a RT less than 200 ms was excluded. When calculating RT, the RT of incorrect trials was also not included. Two types of task switch costs were calculated and used as behavioral outcomes in the study. Global switch costs, also termed general switch costs, were calculated as the differences on reaction time (RT) or accuracy between pure blocks and mixed blocks [27,28]. Local switch costs, also called specific switch costs, were derived only from the mixed blocks, calculated as the differences on RT or accuracy between repetition trials and switch trials [29].

### 2.4. Habitual Physical Activity

The Chinese version of international physical activity questionnaire short-form was administered to assess participants’ daily physical activity level [30]. Metabolic equivalents of task (MET)-minute scores were derived and used as outcomes in this study.

### 2.5. Statistics

Potential differences on participant characteristics between women and men were evaluated by T-Test or chi-squared test. The associations between three measures of adiposity and task-switching performance were analyzed using linear regression modeling. Model assumptions of normality of residuals and detections of multicollinearity were checked by q-q plots and variance inflation factor, respectively. No violations of model assumption were detected. However, a significant adiposity × gender interaction was found in the linear regression models. Therefore, the statistical analyses were stratified by gender. Standardized β coefficients were reported. The statistical analysis was performed using a commercial statistical package STATA 14 (StataCorp LP, College Station, TX, USA), and the null hypothesis was rejected for two-sided values of *p* < 0.05.

## 3. Results

Of the participants, 213 who completed the measurements on cognitive control and adiposity were included in the analyses of the study. Descriptive characteristics were presented in Table 1. Male participants were older, taller, and heavier than female participants (all *p* < 0.05). Males had greater BMI than females (*p* = 0.01). However, males had lower levels of percentage of body fat mass and visceral fat area than females (both *p* < 0.05). According to BMI cut-off points, 13.6% of the participants were overweight or obese. Females were more physically active than males (*p* < 0.05), as indicated by greater MET-minute per week.

Table 2 presents the performance on task switching by gender. Male participants had smaller global switch costs of accuracy (*p* = 0.009), which indicated that the males were better than the females at adapting to the switching task relative to the pure blocks.

Associations of the task-switching performance with the three measures of adiposity were presented in Table 3. Increased BMI (β = 0.25, 95% CI, 0.05 to 0.47, *p =* 0.02), body fat percentage (β = 0.30, 95% CI, 0.05 to 0.54, *p* = 0.02) and visceral fat area (β = 0.26, 95% CI, 0.06 to 0.46, *p* = 0.01) were associated with larger global switch costs of accuracy in women, with adjustment for age and daily physical activity. In men, body fat percentage (β = 0.31, 95% CI, 0.07 to 0.54, *p* = 0.01) and visceral fat area (β = 0.20, 95% CI, 0.02 to 0.38, *p* = 0.03) were associated with larger local switch costs of RT. The results indicated that elevated adiposity was significantly correlated with worse performance on some aspects of cognitive control in young women and men.

## 4. Discussion

In this study, the associations of cognitive control with three measures of adiposity were investigated in Chinese young adults. In women, increased BMI, body fat percentage, and visceral fat area were associated with higher global switch costs. In men, increased body fat percentage and visceral fat area were associated with higher local switch costs. The findings indicated that elevated adiposity was associated with worse performance on the task-switching paradigm.

In women, the higher levels of measures of adiposity were correlated to higher levels of global accuracy switch costs of the task-switching paradigm. Global switch costs are thought to reflect the ability of maintaining multiple tasks and coordinating task sets [31,32]. Although different cognitive tasks were used, our results are generally in accordance with the findings from previous studies in children [33,34] and young adults [20,21]. Catoira et al. [21] demonstrated that obese young women had worse inhibitory control compared with normal weight individuals. Song et al. [20] showed that obese and low-fitness college students had worse inhibitory control than normal weight and high-fitness individuals. These two studies only compared obese individuals with normal weight controls. However, in our study, we observed a linear relationship with adiposity and performance on the cognitive control task in women. Most of the participants in our study were normal weight individuals. It is less likely that the observed associations were only driven by the overweight and obese individuals. Therefore, it is possible that even in non-obese young women, increased body fat mass is associated with poorer cognitive control. From a perspective of public health, the findings have important implications, which indicate that the negative associations between cognitive control and adiposity may exist in young adults even with normal weight. Moreover, our findings extended the current literature by showing that adiposity is also associated with worse performance of the task-switching paradigm, which requires working memory and cognitive flexibility. In addition, the current study also observed a trend (near significant) of negative associations between adiposity and local switch costs in women. The reasons for these unexpected trends are not clear. More studies are warranted to validate the findings and clarify the possible reasons.

In men, BMI was not associated with the measures of cognitive control. However, higher levels of body fat percentage and visceral fat area were associated with higher local RT switch costs. Local switch costs reflect the efficiency of cognitive processes concerned with initiation and execution of actual task set shift [28]. Our findings in men were generally supported by a recent study in older adults by Ntlholang et al. [35], who showed that central adiposity was a stronger predictor of worse cognitive function than BMI. However, another study by Bove et al. [36] showed that adiposity was not linked with executive domains of cognitive function in young men. However, they found that total body fat was correlated with worse function in visual memory and visuospatial skills. It is worth noting that our study differed from Bove’s [36] study in terms of participants. All the participants in their study were with a BMI larger than 25 kg/m^2^. In our study, the participants were randomly recruited. Furthermore, the current study demonstrated that higher body fat percentage and visceral fat, rather than BMI, were associated with worse cognitive control in men. It may be attributed to the limitation of BMI, since BMI fails to distinguish between body fat mass and lean mass [37].

Interestingly, in the present study, elevated adiposity was associated with different indicators of task-switching performance (i.e., global switch costs and local switch costs) in women and men, respectively. It is possible that excessive adiposity may exact gender-specific influences on cognitive control in women and men, since the distinct constructs were reflected by global switch costs and local switch costs, respectively [28]. However, the underlying reasons were not clear. Evidence has demonstrated the differences in neural organization of task-switching process between females and males [38], which may explain part of the gender-specific findings. More studies are required to further confirm the findings and clarify the potential mechanisms.

Some limitations of the study should be acknowledged. First, this study was a cross-sectional study. The design of the study made it impossible to infer potential causality. Therefore, no causal inference can be made based on the observed associations in the current study. Elevated adiposity may lead to poorer performance on cognitive control. Meanwhile, it is also possible that poor cognitive control lead to increased risk of being obese or overweight. Indeed, recent studies have demonstrated that poor cognitive control influences obesity-related behaviors, such as uncontrolled eating and consumption of high-energy food in adolescents and young adults [39,40]. Second, a widely used task-switching paradigm was administered in this study. The measure cannot reflect participants’ overall cognitive control. However, the use of a task-switching paradigm ensured the high comparability of our findings. Third, the use of BMI may be another limitation, since, as aforementioned, it cannot distinguish between body fat mass and lean mass. In addition, the participants were recruited from one university and were about the same age, which may limit the generalization of the results. Though this group of participants is an important population to study, more studies with diverse sample are required to confirm whether adiposity is linked with cognitive control in young adulthood.

## 5. Conclusions

The results demonstrated that elevated adiposity was associated with larger global switch costs on the task-switching paradigm in women. In men, elevated adiposity was associated with larger local switch costs. The findings indicated that elevated adiposity was associated with worse performance on some aspects of cognitive control in young adults.

## Figures and Tables

**Table 1:** Baseline characteristics of participants.

Variables	Women (N = 98)	Men (N = 115)	All (N = 213)	*p* For Gender
Age (years)	18.6 ± 0.9	19.3 ± 1.1 *	19.0 ±1.0	<0.001
Height (cm)	163.3 ± 7.0	174.7 ± 5.7 *	169.4 ± 8.5	<0.001
Weight (kg)	54.8 ± 7.2	65.9 ± 11.3 *	60.8 ± 11.1	<0.001
BMI (kg/m^2^)	20.6 ± 2.4	21.5 ± 3.2 *	21.1 ± 2.9	0.01
Overweight and obesity (%)	9.2%	17.4%	13.6%	0.08
Body fat mass (%)	27.1 ± 5.1	16.9 ± 5.5 *	21.6 ± 7.3	<0.001
Visceral fat area (cm^2^)	61.7 ± 21.5	44.9 ± 25.1 *	52.7 ± 49.3	<0.001
IPAQ (MET-minute/week)	2408.7 ± 1608.1	1827.4 ± 1404.8	2097.4 ± 1526.9	0.006

Abbreviation: BMI, body mass index; IPAQ, international physical activity questionnaire; MET, metabolic equivalent of task. Data are expressed as mean ± standard deviation (SD). Overweight and obesity were defined based on BMI.

**Table 2:** Summary of task switching performance by gender.

	Women (n = 98)	Men (n = 115)	Total (n = 213)	*p* for Gender
RT of pure blocks (ms)	328.5 ± 61.9	325.5 ± 63.5	326.8 ± 62.6	0.73
Accuracy of pure blocks (%)	95.3 ± 4.0	94.5 ± 5.3	94.9 ± 4.8	0.23
RT of mixed blocks (ms)	563.5 ± 140.4	574.6 ± 156.3	569.5 ± 148.9	0.59
Accuracy of mixed blocks (%)	91.0 ± 6.5	92.1 ± 5.3	91.6 ± 5.9	0.17
Global switch costs-RT (ms)	235.0 ± 116.2	249.2 ± 130.5	242.6 ± 124.0	0.41
Global switch costs-Accuracy (%)	4.3 ± 4.7	2.4 ± 5.6	3.3 ± 5.3	0.009
Local switch costs-RT (ms)	52.2 ± 69.5	60.6 ± 66.3	56.7 ± 67.8	0.37
Local switch costs-Accuracy (%)	3.2 ± 4.1	2.6 ± 4.8	2.9 ± 4.5	0.34

Abbreviations: RT, reaction time. Data are expressed as mean ± standard deviation (SD).

**Table 3:** Associations of task switching performance with measures of adiposity.

	BMI		Body Fat Percentage		Visceral Fat Area	
	Standardized β (95% CI)	*p*	Standardized β (95% CI)	*p*	Standardized β (95% CI)	*p*
RT of pure blocks						
Women	−0.10 (−0.36, 0.15)	0.43	0.09 (−0.21, 0.39)	0.53	0.04 (−0.20, 0.29)	0.72
Men	0.03 (−0.16, 0.22)	0.77	0.03 (−0.25, 0.30)	0.86	0.03 (−0.17, 0.24)	0.74
Accuracy of pure blocks						
Women	−0.11 (−0.31, 0.09)	0.27	−0.04 (−0.28, 0.20)	0.75	−0.07 (−0.26, 0.13)	0.51
Men	−0.09 (−0.30, 0.11)	0.38	−0.14 (−0.43, 0.15)	0.34	−0.12 (−0.33, 0.10)	0.29
RT of mixed blocks						
Women	−0.04 (−0.28, 0.19)	0.71	−0.10 (−0.38, 0.18)	0.50	−0.01 (−0.24, 0.22)	0.96
Men	0.11 (−0.07, 0.30)	0.23	0.08 (−0.19, 0.34)	0.57	0.11 (−0.08, 0.31)	0.26
Accuracy of mixed blocks						
Women	−0.22 (−0.46, 0.02)	0.07	−0.20 (−0.49, 0.08)	0.16	−0.20 (−0.43, 0.03)	0.08
Men	−0.01 (−0.18, 0.16)	0.90	0.06 (−0.18, 0.30)	0.63	0.02 (−0.16, 0.20)	0.86
Global switch cost-RT						
Women	−0.003 (−023, 0.23)	0.98	−0.15 (−0.42, 0.12)	0.27	−0.03 (−0.25, 0.19)	0.78
Men	0.08 (−0.09, 0.26)	0.35	0.09 (−0.16, 0.35)	0.47	0.10 (−0.09, 0.30)	0.29
Global switch cost-Accuracy						
Women	0.25 (0.05, 0.47)	0.02	0.30 (0.05, 0.54)	0.02	0.26 (0.06, 0.46)	0.01
Men	−0.09 (−0.27, 0.09)	0.32	−0.21 (−0.48, 0.50)	0.11	−0.16 (−0.36, 0.04)	0.11
Local switch cost-RT						
Women	−0.19 (−0.44, 0.05)	0.12	−0.27 (−0.56, 0.02)	0.07	−0.23 (−0.47, 0.004)	0.06
Men	0.11 (−0.05, 0.28)	0.18	0.31 (0.07, 0.54)	0.01	0.20 (0.02, 0.38)	0.03
Local switch cost-Accuracy						
Women	0.02 (−0.21, 0.25)	0.89	−0.14 (−0.41, 0.13)	0.30	−0.04 (−0.27, 0.18)	0.69
Men	−0.07 (−0.26, 0.11)	0.43	−0.17 (−0.43, 0.10)	0.21	−0.11 (−0.31, 0.09)	0.30

Abbreviations: RT, reaction time. All analyses were adjusted for age and physical activity. Data are expressed as standardized β and 95% confidence interval (95% CI).

## References

[1] Di Cesare M., Bentham J., Stevens G.A., Zhou B., Danaei G., Lu Y., Bixby H., Cowan M.J., Riley L.M., Hajifathalian K. (2016). Trends in adult body-mass index in 200 countries from 1975 to 2014: A pooled analysis of 1698 population-based measurement studies with 19.2 million participants. Lancet.

[2] Afshin A., Forouzanfar M.H., Reitsma M.B., Sur P., Estep K., Lee A., Marczak L., Mokdad A.H., Moradi-Lakeh M., GBD 2015 Obesity Collaborators (2017). Health effects of overweight and obesity in 195 Countries over 25 Years. N. Engl. J. Med..

[3] Tian Y., Jiang C., Wang M., Cai R., Zhang Y., He Z., Wang H., Wu D., Wang F., Liu X. (2016). BMI, leisure-time physical activity, and physical fitness in adults in China: Results from a series of national surveys, 2000−14. Lancet Diabetes Endocrinol..

[4] Wormser D., Kaptoge S., Di Angelantonio E., Wood A.M., Pennells L., Thompson A., Sarwar N., Kizer J.R., Lawlor D.A., Nordestgaard B.G. (2011). Separate and combined associations of body-mass index and abdominal adiposity with cardiovascular disease: Collaborative analysis of 58 prospective studies. Lancet.

[5] Lu Y., Hajifathalian K., Ezzati M., Woodward M., Rimm E.B., Danaei G. (2014). Metabolic mediators of the effects of body-mass index, overweight, and obesity on coronary heart disease and stroke: A pooled analysis of 97 prospective cohorts with 1.8 million participants. Lancet.

[6] Wade K.H., Carslake D., Sattar N., Davey Smith G., Timpson N.J. (2018). BMI and Mortality in UK Biobank: Revised estimates using mendelian randomization. Obesity (Silver Spring).

[7] Wade K.H., Chiesa S.T., Hughes A.D., Chaturvedi N., Charakida M., Rapala A., Muthurangu V., Khan T., Finer N., Sattar N. (2018). Assessing the causal role of body mass index on cardiovascular health in young adults: Mendelian randomization and recall-by-genotype analyses. Circulation.

[8] O’Brien P.D., Hinder L.M., Callaghan B.C., Feldman E.L. (2017). Neurological consequences of obesity. Lancet Neurol..

[9] Anstey K.J., Cherbuin N., Budge M., Young J. (2011). Body mass index in midlife and late-life as a risk factor for dementia: A meta-analysis of prospective studies. Obes. Rev..

[10] Loef M., Walach H. (2013). Midlife obesity and dementia: Meta-analysis and adjusted forecast of dementia prevalence in the United States and China. Obesity (Silver Spring).

[11] Sabia S., Kivimaki M., Shipley M.J., Marmot M.G., Singh-Manoux A. (2009). Body mass index over the adult life course and cognition in late midlife: The whitehall II cohort study. Am. J. Clin. Nutr..

[12] Strachan M.W., Reynolds R.M., Marioni R.E., Price J.F. (2011). Cognitive function, dementia and type 2 diabetes mellitus in the elderly. Nat. Rev. Endocrinol..

[13] Nameni G., Farhangi M.A., Hajiluian G., Shahabi P., Abbasi M.M. (2017). Insulin deficiency: A possible link between obesity and cognitive function. Int. J. Dev. Neurosci..

[14] Cunningham C., Campion S., Lunnon K., Murray C.L., Woods J.F., Deacon R.M., Rawlins J.N., Perry V.H. (2009). Systemic inflammation induces acute behavioral and cognitive changes and accelerates neurodegenerative disease. Biol. Psychiatry.

[15] Willette A.A., Kapogiannis D. (2015). Does the brain shrink as the waist expands?. Ageing Res. Rev..

[16] Nolan P.B., Carrick-Ranson G., Stinear J.W., Reading S.A., Dalleck L.C. (2017). Prevalence of metabolic syndrome and metabolic syndrome components in young adults: A pooled analysis. Prev. Med. Rep..

[17] Schippling S., Ostwaldt A.C., Suppa P., Spies L., Manogaran P., Gocke C., Huppertz H.J., Opfer R. (2017). Global and regional annual brain volume loss rates in physiological aging. J. Neurol..

[18] Diamond A. (2013). Executive functions. Annu. Rev. Psychol..

[19] Allan J.L., McMinn D., Daly M. (2016). A Bidirectional relationship between executive function and health behavior: Evidence, implications, and future directions. Front. Neurosci..

[20] Song T.F., Chi L., Chu C.H., Chen F.T., Zhou C., Chang Y.K. (2016). Obesity, cardiovascular fitness, and inhibition function: An electrophysiological study. Front. Psychol..

[21] Catoira N.P., Tapajóz F., Allegri R.F., Lajfer J., Rodríguez Cámara M.J., Iturry M.L., Castaño G.O. (2016). Obesity, metabolic profile, and inhibition failure: Young women under scrutiny. Physiol. Behav..

[22] Ogawa H., Fujitani K., Tsujinaka T., Imanishi K., Shirakata H., Kantani A., Hirao M., Kurokawa Y., Utsumi S. (2011). InBody 720 as a new method of evaluating visceral obesity. Hepatogastroenterology.

[23] Furstenberg A., Davenport A. (2011). Assessment of body composition in peritoneal dialysis patients using bioelectrical impedance and dual-energy x-ray absorptiometry. Am. J. Nephrol..

[24] Zhou B.F. (2002). Predictive values of body mass index and waist circumference for risk factors of certain related diseases in Chinese adults—Study on optimal cut-off points of body mass index and waist circumference in Chinese adults. Biomed. Environ. Sci..

[25] Kamijo K., Takeda Y. (2010). Regular physical activity improves executive function during task switching in young adults. Int. J. Psychophysiol..

[26] Rogers R.D., Monsell S. (1995). Costs of a predictible switch between simple cognitive tasks. J. Exp. Psychol. Gen..

[27] Peng A., Kirkham N.Z., Mareschal D. (2018). Task switching costs in preschool children and adults. J. Exp. Child. Psychol..

[28] Kray J., Lindenberger U. (2000). Adult age differences in task switching. Psychol. Aging.

[29] Themanson J.R., Hillman C.H., Curtin J.J. (2006). Age and physical activity influences on action monitoring during task switching. Neurobiol. Aging.

[30] Fan M., Lyu J., He P. (2014). Chinese guidelines for data processing and analysis concerning the International Physical Activity Questionnaire. Zhonghua Liu Xing Bing Xue Za Zhi.

[31] Bojko A., Kramer A.F., Peterson M.S. (2004). Age equivalence in switch costs for prosaccade and antisaccade tasks. Psychol. Aging.

[32] Rizzo M., Anderson S., Fritzsch B. (2018). The Wiley Handbook on the Aging Mind and Brain.

[33] Huang T., Tarp J., Domazet S.L., Thorsen A.K., Froberg K., Andersen L.B., Bugge A. (2015). Associations of adiposity and aerobic fitness with executive function and math performance in danish adolescents. J. Pediatr..

[34] Kamijo K., Khan N.A., Pontifex M.B., Scudder M.R., Drollette E.S., Raine L.B., Evans E.M., Castelli D.M., Hillman C.H. (2012). The relation of adiposity to cognitive control and scholastic achievement in preadolescent children. Obesity (Silver Spring).

[35] Ntlholang O., McCarroll K., Laird E., Molloy A.M., Ward M., McNulty H., Hoey L., Hughes C.F., Strain J.J., Casey M. (2018). The relationship between adiposity and cognitive function in a large community-dwelling population: Data from the Trinity Ulster Department of Agriculture (TUDA) ageing cohort study. Br. J. Nutr..

[36] Bove R.M., Gerweck A.V., Mancuso S.M., Bredella M.A., Sherman J.C., Miller K.K. (2016). Association between adiposity and cognitive function in young men: Hormonal mechanisms. Obesity (Silver SpringMd).

[37] Okorodudu D.O., Jumean M.F., Montori V.M., Romero-Corral A., Somers V.K., Erwin P.J., Lopez-Jimenez F. (2010). Diagnostic performance of body mass index to identify obesity as defined by body adiposity: A systematic review and meta-analysis. Int. J. Obes. (Lond.).

[38] Kuptsova S.V., Ivanova M.V., Petrushevsky A.G., Fedina O.N., Zhavoronkova L.A. (2015). Sex differences in visual task switching (an fMRI Study). Fiziol. Cheloveka.

[39] Maayan L., Hoogendoorn C., Sweat V., Convit A. (2011). Disinhibited eating in obese adolescents is associated with orbitofrontal volume reductions and executive dysfunction. Obesity (Silver Spring).

[40] Limbers C.A., Young D. (2015). Executive functions and consumption of fruits/vegetables and high saturated fat foods in young adults. J. Health Psychol..

